# Warburg Effect as a Novel Mechanism for Homocysteine-Induced Features of Age-Related Macular Degeneration

**DOI:** 10.3390/ijms24021071

**Published:** 2023-01-05

**Authors:** Yara A. Samra, Yusra Zaidi, Pragya Rajpurohit, Raju Raghavan, Lun Cai, Ismail Kaddour-Djebbar, Amany Tawfik

**Affiliations:** 1 Department of Biochemistry, Faculty of Pharmacy, Mansoura University, Mansoura 35516, Egypt; 2 Department of Oral Biology and Diagnostic Sciences, Dental College of Georgia, Augusta University, Augusta, GA 30912, USA; 3 Department of Pharmacology, Medical College of Georgia, Augusta University, Augusta, GA 30912, USA; 4 Department of Physiology, Medical College of Georgia, Augusta University, Augusta, GA 30912, USA; 5 Charlie Norwood VA Medical Center, One Freedom Way, Augusta, GA 30904, USA; 6 Eye Research Institute, Oakland University, Rochester, MI 48309-4479, USA; 7 Eye Research Center (OUWB)/ERC, William Beaumont School of Medicine, Royal Oak, MI 48309-4479, USA

**Keywords:** N-methyl-D-aspartate receptor, homocysteine, age-related macular degeneration, blood retinal barrier, cystathionine-β—synthase, mouse

## Abstract

Age-related macular degeneration (AMD) is a major cause of blindness. Recent studies have reported impaired glycolysis in AMD patients with a high lactate/pyruvate ratio. Elevated homocysteine (Hcy) (Hyperhomocysteinemia, HHcy) was observed in several clinical studies, reporting an association between HHcy and AMD. We established the effect of HHcy on barrier function, retinal pigment epithelium (RPE) structure, and induced choroidal neovascularization (CNV) in mice. We hypothesize that HHcy contributes to AMD by inducing a metabolic switch in the mitochondria, in which cells predominantly produce energy by the high rate of glycolysis, or “Warburg”, effect. Increased glycolysis results in an increased production of lactate, cellular acidity, activation of angiogenesis, RPE barrier dysfunction, and CNV. Evaluation of cellular energy production under HHcy was assessed by seahorse analysis, immunofluorescence, and western blot experiments. The seahorse analysis evaluated the extracellular acidification rate (ECAR) as indicative of glycolysis. HHcy showed a significant increase in ECAR both in vivo using (Cystathionine β-synthase) *cbs^+/−^* and *cbs^−/−^* mice retinas and in vitro (Hcy-treated ARPE-19) compared to wild-type mice and RPE cells. Moreover, HHcy up-regulated glycolytic enzyme (Glucose transporter-1 (GlUT-1), lactate dehydrogenase (LDH), and hexokinase 1 (HK1)) in Hcy-treated ARPE-19 and primary RPE cells isolated from *cbs^+/+^*, *cbs^+/−^*, and *cbs^−/−^* mice retinas. Inhibition of GLUT-1 or blocking of N-methyl-D-aspartate receptors (NMDAR) reduced glycolysis in Hcy-treated RPE and improved albumin leakage and CNV induction in Hcy-injected mice eyes. The current study suggests that HHcy causes a metabolic switch in the RPE cells from mitochondrial respiration to glycolysis during AMD and confirms the involvement of NMDAR in this process. Therefore, targeting Glycolysis or NMDAR could be a novel therapeutic target for AMD.

## 1. Introduction

Age-related macular degeneration (AMD) is the most commonly known cause of vision loss in people over 60 [1]. AMD mainly affects their central vision, in which patients cannot see things directly in front of them. There are two types of disease: dry-type AMD, which leads to gradual visual impairment, and wet-type AMD, which tends to cause a rapid loss of vision [1,2]. Eleven million new cases are diagnosed in the United States (USA) every year [3]. Therefore, AMD is the main cause of visual disability in developed countries and the third globally [4]. The low quality of life of AMD patients and high treatment costs make it urgent to find a new therapy for AMD. Anti-(vascular endothelial growth factor) VEGF therapy has made important progress in treating the wet form of AMD by targeting the abnormally formed blood vessels, but these patients still exhibit a progression of the disease and do not regain vision despite treatments. Over the last fifteen years, Hyperhomocysteinemia (HHcy) has gained special consideration in many AMD studies, which suggest an association between HHcy and AMD [5,6,7,8]. Furthermore, Over the past several years, we have conducted experiments aimed to address this issue. Earlier studies reported a direct impact of excess homocysteine (Hcy) on (retinal pigment epithelium) RPE structure, barrier function, and induced choroidal neovascularization (CNV) in mice [9], and subsequent experiments reported that HHcy not only increased hypoxia-inducible factor 1 (HIF-1α) and VEGF levels in retinas [10,11], but also angiogenic potential of retinal endothelial cells in vitro and in vivo [10,12,13]. These findings were followed by many mechanistic studies that showed that HHcy activates oxidative stress [12], endoplasmic reticulum (ER) stress [14], inflammation [15], and epigenetic modifications [16]. Recently, we reported activation of the N-methyl-d-aspartate (NMDA) receptor in retinal endothelial cells [17] and in RPE [11] cells as a suggested mechanism of HHcy-induced retinal dysfunction. Our goal is to understand the exact mechanism by which HHcy participates in the pathogenesis of AMD; accordingly, we can propose Hcy as a marker and therapeutic target for AMD. 

Photoreceptor growth and survival depend on glucose transport through the RPE cells. Glucose from choroidal circulation has to traverse the RPE cytoplasm to get to the outer retina. Therefore, to satisfy the retina’s large requirement for glucose, retinal RPE expresses high levels of glucose transporters [18]. Glucose transporter-1 (GLUT-1) was reported as a dominant glucose transporter in RPE, which is located mainly on the apical and basolateral surfaces of RPE plasma membranes [19]. Its activation enhances intracellular glucose transport in retinal cells [20]. RPE mitochondrial metabolism is critical for retinal viability, but the RPE cells cannot consume too much glucose for their own metabolism because the retina could starve.

Under hypoxic conditions, the transcription factor HIF-1α was reported to enhance glycolysis, leading to faster consumption of glucose in the RPE than normal, and photoreceptor degeneration [21,22]. Meanwhile, the effect of glucose and glucose metabolism on HIF-1α stability served as a feedback mechanism, whereby HIF-1α accelerated the expression and activation of GLUT1 and induced glucose uptake and glycolysis which in turn induced HIF-1α degradation [23].

Moreover, Vollrath and colleagues reported the importance of mitochondrial respiration in the RPE. Genetic manipulation of a key protein essential for the maintenance of mitochondrial DNA disrupted RPE mitochondria, which was followed by mitochondrial loss. The mitochondrial loss altered the metabolism of the RPE cells and caused degeneration of retinal photoreceptors [22,23]. Alongside serving as the outer blood–retinal barrier (BRB), RPE is the major transport pathway for the exchange of metabolites, nutrients, and ions between choroidal blood vessels and the neural retina [24]. Nutrients derived from different metabolic pathways are oxidized to produce cellular energy in the form of ATP [25]. Energy is generated in two ways: glycolytic (non-oxidative breakdown) and oxidative phosphorylation when oxygen is present in cells. One glucose molecule yields only two molecules of ATP via the glycolytic pathway but 36 molecules of ATP via the oxidative pathway [26]. Collected information from recent studies and the findings in the current study suggest that the switch from mitochondrial metabolism to glycolysis in the presence of oxygen (known as aerobic glycolysis or the Warburg effect) induces RPE cells to increase their glucose consumption. The RPE then could not provide sufficient glucose to the outer retina. Furthermore, accelerated glucose consumption via glycolysis leads to the accumulation of lactate with increased cellular acidity and oxidative stress. Accumulating evidence from recent studies of retinal energy metabolism suggests that the retina and the RPE cells are dependent on each other and work together as a metabolic ecosystem, assuming complementary metabolic roles that are essential for their survival and for their capacities to perform their specialized functions. 

**Figure 1:**
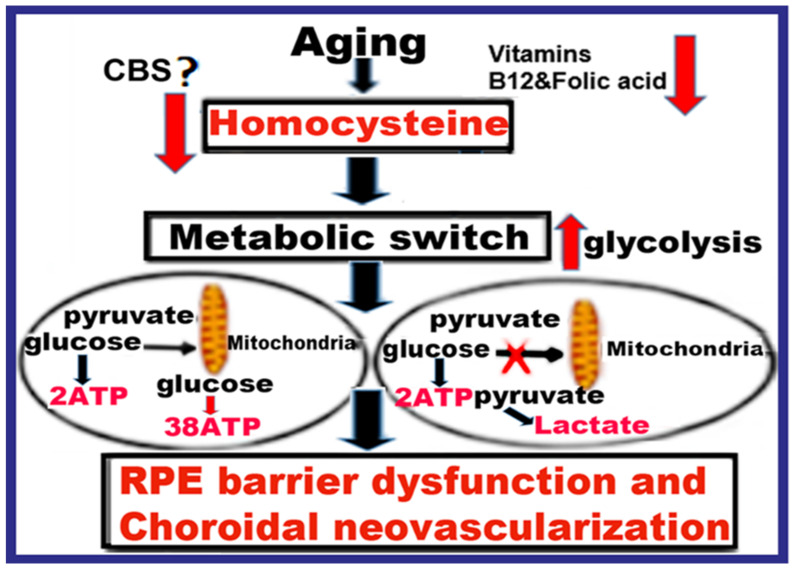

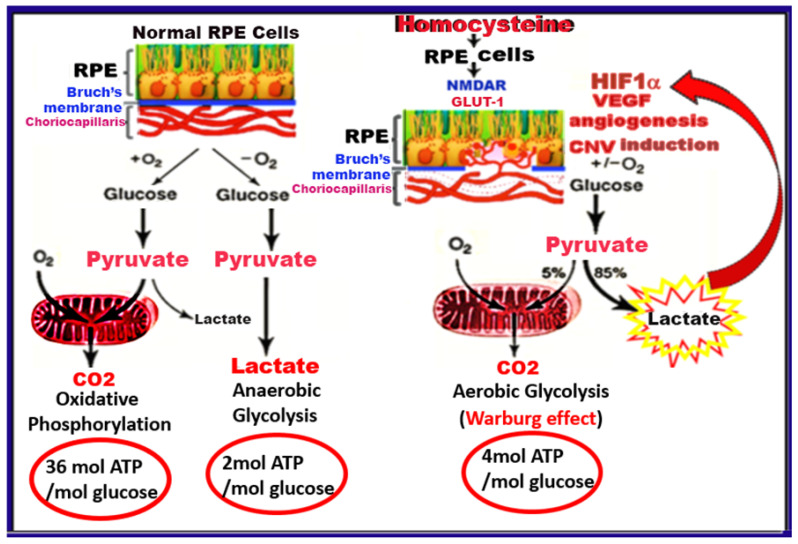
The schematic diagram for the research hypothesis. Schematic representation of the study’s hypothesis of HHcy involvement in glycolysis, RPE dysfunction, and CNV induction via up-regulation of GLUT-1 in RPE cells, inducing a Warburg-like effect in which cells predominantly produce energy by a high rate of glycolysis, rather than by a comparatively low rate of glycolysis followed by oxidation of pyruvate in mitochondria.

Recent clinical studies reported impaired glycolysis in AMD patients with a high lactate/pyruvate ratio and suggest that increased lactate levels may be implicated in the pathogenesis of AMD [27]. Previously, we reported that Hcy participates in alterations of mitochondrial dynamics in retinal ganglion cells [28]. Other studies have reported Hcy induces similar metabolic changes in retinal Müller cells [29] and immune cells [30]. Thus, we hypothesize that HHcy contributes to AMD via the up-regulation of Glucose transporter-1 (GLUT-1) in RPE cells, inducing a Warburg-like effect in which cells predominantly produce energy by a high rate of glycolysis, rather than by a comparatively low rate of glycolysis followed by oxidation of pyruvate in mitochondria as in most normal cells [31]. Furthermore, we propose the involvement of N-methyl-D-aspartate receptor (NMDAR) in this process. Figure 1 is schematic representation of the hypothesis.

## 2. Results

### 2.1. Homocysteine Activates Glucose Transporter 1 (GLUT-11) in Retinal RPE Cells

**Figure 2:**
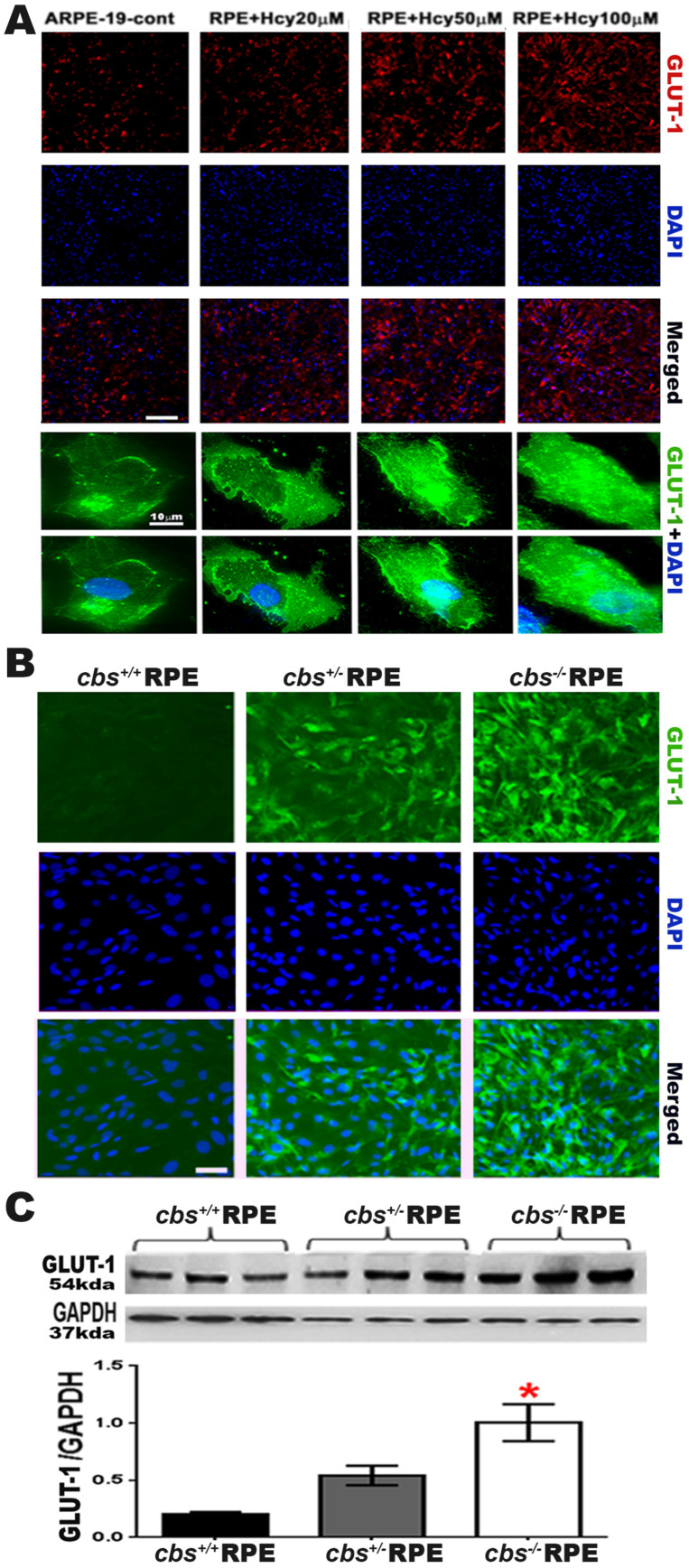
Homocysteine activates GLUT1 in RPE cells. (**A**) Upper part: immunofluorescence staining for GLUT1 in ARPE-19 (red) treated with different concentrations of Hcy (20, 50, and 100 μM). High magnification image showing that Hcy significantly increased GLUT1 expression in ARPE cells in a dose-dependent manner. Lower part: high magnification image immunofluorescence staining for GLUT1 in ARPE-19 (green) treated with different concentrations of Hcy (20, 50, and 100 μM). (**B**) Immunofluorescence staining for GLUT1 in primary RPE cells isolated from wild-type *cbs^+/+^*, *cbs^+/−^* and *cbs^−/−^* mice (green) with nuclear staining (DAPI, blue). (**C**) Western blot of GLUT1 expression in primary RPE cells isolated from *cbs^+/+^*, *cbs^+/−^* and *cbs^−/−^* mice. GADPH was used as a loading control * *p* < 0.05 as compared to WT-RPE and *cbs*^+/−^ mice. The data show that Hcy significantly increased GLUT1 expression of RPE cells both in vivo and ex-vivo. Scale bar: 50 μm (A upper part &B) and 10 μm (A lower part). * *p* < 0.05 compared to *cbs ^+/+^* and *cbs ^+/−^*.

GLUT1 is the primary glucose transporter that enables glucose transport across outer the BRB formed by the RPE [19]. It is expressed mainly and at a high level in the apical and basolateral membranes of the RPE of the outer BRB and in the luminal and abluminal membranes of the endothelial cells of the inner BRB [20,32]. To study the effect of Hcy on GLUT1 activation, the GLUT1 expression was evaluated in primary RPE cells isolated from a mouse model of HHcy (wild-type *cbs^+/+^*, *cbs^+/−^* and *cbs^−/−^* mice, representing the normal level of Hcy, mild/moderate HHcy, and sever HHcy). Immunofluorescence staining of retinal frozen sections for GLUT1 showed increased GLUT1 expression by Hcy in ARPE cells in a dose-dependent manner (Figure 2A, upper part red, low magnification and lower part, green, high magnification). Similarly, GLUT1 expression was increased in the *cbs^+/−^* and *cbs^−/−^* RPE cells (Figure 2B, green), and this up-regulation of GLUT1 expression is associated with more prominent Hcy in the *cbs^−/−^* mice Up-regulation of GLUT1 expression by HHcy was further confirmed by western blot analysis in the primary RPE cells (Figure 2C). GLUT1 expression was significantly increased in *cbs^−/−^* mice. Scale bar: 50 μm. * *p* < 0.05.

### 2.2. Homocysteine Activates Glycolytic Enzymes in RPE Cells

Glycolytic enzymes are a set of enzymes located in the sarcoplasmic reticulum. They convert glucose-6-phosphate and nicotinamide adenine dinucleotides (NAD+) to pyruvate and NADH resulting in the production of two molecules of ATP. Hexokinase 1 (HK1) is an intracellular enzyme, the initial step and rate-limiting enzyme of glycolysis that catalyzes the phosphorylation of glucose by ATP to glucose-6-P [33,34]. Previously, we reported that HHcy disrupts retinal pigment epithelial barrier function and induces choroidal neovascularization (CNV) [9,35]. The current study proposed that HHcy activates GLUT1 in the RPE cells with a subsequent increase in glycolysis. Thus, we evaluated HK1 and the Lactate dehydrogenase (LDH) enzyme, which catalyzes the last step in glycolysis, resulting in L-lactate conversion to pyruvate [34,36].

**Figure 3:**
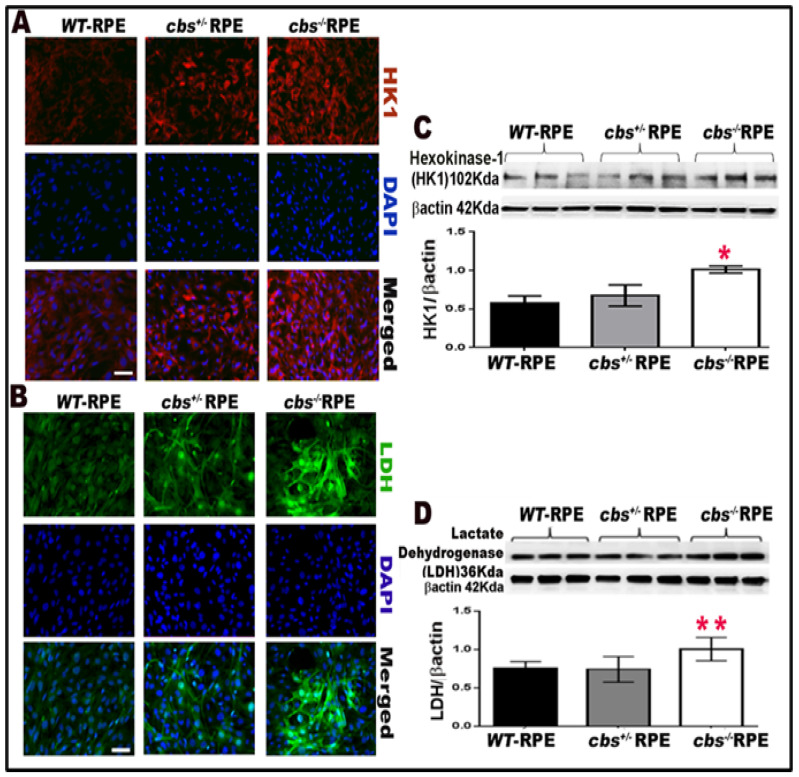
Homocysteine promotes glycolysis by activating glycolytic enzymes. (**A**) Immunofluorescence staining for HK-1 (red) and nuclear staining (DAPI, blue) for primary RPE cells isolated from *cbs^+/+^ cbs^+/−^*, and *cbs^−/−^* mice. (**B**) Immunofluorescence staining for LDH (green) and nuclear staining (DAPI, blue) for RPE cells isolated from *cbs^+/+^* andd *cbs^+/−^* mice. (**C**) Western blot of HK-1 expression in primary RPE cells isolated from mice model of elevated Hcy (wild-type *cbs^+/+^*, *cbs^+/−^*, and *cbs^−/−^*) showing a significant increase in HK-1 expression in mice with marked HHcy. β actin was used as loading control. (**D**) Western blot of LDH expression in primary RPE cells isolated from mice model of elevated Hcy (wild-type *cbs^+/+^*, *cbs^+/−^*, and *cbs^−/−^*) showing a significant increase in LDH expression in mice with marked HHcy. βactin was used as a loading control. Scale bar: 50 μm. * *p* < 0.05, ** *p* < 0.01 compared to WT and *cbs^+/−^* RPE.

To further study the activation of glycolysis in the RPE by Hcy, we evaluated the enzymes involved in glycolysis’s initial step (HKI-1) and last steps (LDH). Hexokinase and L-lactate dehydrogenase enzymes were evaluated in primary RPE cells isolated from wild-type *cbs^+/+^*, *cbs^+/−,^*, and *cbs^−/−^* mice, as evaluated by immunofluorescence (Figure 3A,B) and western blot analysis (Figure 3C,D). HKI-1 and LDH levels were significantly increased in both the *cbs^+/−^* (represent mild/moderate HHcy) and *cbs^−/−^* mice RPE cells (represent severe HHcy); this was evident in immunofluorescence staining and western blot analysis compared to wild-type *cbs^+/+^* retinas. These data confirm the activation of glycolysis by elevated Hcy. Scale bar: 50 μm. * *p* < 0.05, ** *p* < 0.01.

### 2.3. Effect of Glut-1 Inhibition on Hcy-Induced BRB Dysfunction 

**Figure 4:**
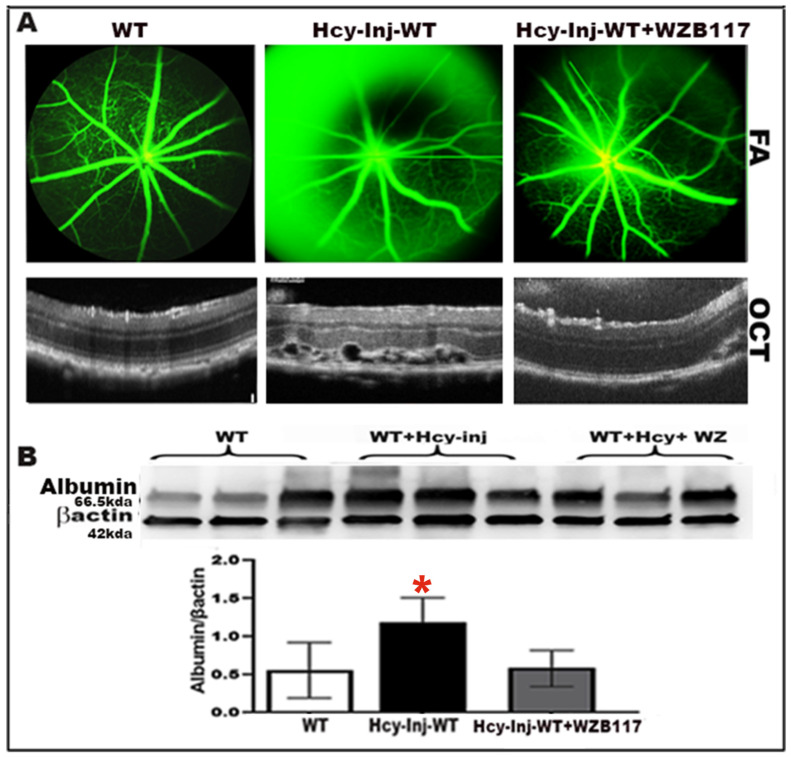
Inhibition of glycolysis protects the retina from Homocysteine induced blood–retinal barrier (BRB) dysfunction and choroidal neovascularization (CNV). C57BL6 mice with/without Hcy-thiolactone intravitreal injection in the presence or absence of intraperitoneal (IP) injection of GLUT1 pharmacological inhibitor WZB117 were evaluated 72 h after Hcy injection by (**A**) FA evaluation (upper panel), showing normal well-formed vessels in WT mouse; however, Hcy-injected mouse retinas show obvious vascular leakage manifesting as diffused hyperfluorescence, which was markedly reduced after blocking GLUT1 with WZB117. The OCT examination (lower panel) shows a normal appearance in WT mouse retinas, as well as obvious interruption of retinal morphology with notable hyporeflective subretinal lucency, focal hyperreflective spots, and choroidal neovascularization in Hcy-injected mice retinas. Blocking GLUT1 1 was able to reduce the retinal disruption, and it improved retinal structure and CNV induction after Hcy injection in WT-type mice (n = 6 mice/group. (**B**) Western blot evaluation of albumin leakage in mouse retinas after perfusion of mice with PBS solution. Blocking of GLUT1 was able to decrease the albumin leakage in the retinas, which was significantly increased in the Hcy-injected mice eyes. * *p* < 0.05 as compared to WT and WZB117 injected mice.

We wanted to examine whether GLUT1 inhibition will rescue the retina from Hcy-induced blood–retinal barrier (BRB) disruption and CNV induction. A functional in vivo study was conducted, using Fluorescein angiography (FA) and optical coherence tomography (OCT) examination in living mice. FA and OCT were used to evaluate vascular leakage, retinal morphology, and CNV induction in living mice. Three groups of mice were used for this purpose at age ~6–8 weeks old (6 mice/group). Mice were subjected to FA and OCT evaluation (wild-type (C57-BL6)) injected intraperitoneal with specific GLUT1 inhibitor WZB117 (10 mg/kg) [36] (to exclude any toxic effect of WZB117, it was injected IP without Hcy-injection followed by evaluation of retinal structure and function) compared to wild-type mice injected intravitreally with Hcy (200 μM) in the presence/absence intraperitoneal of WZB117 (IP, twice in two days before Hcy intravitreal injection). The OCT and FA evaluations were performed 72 h after intravitreal injection of Hcy. FA examination (Figure 4A, upper panel) showed increased fluorescein leakage and disrupted retinal morphology in Hcy-injected wild-type mice compared to wild-type control mice, suggesting decreased integrity of retinal blood vessels and BRB impairment by Hcy, and confirming our previous publications [9,11,16,17,37]. While OCT evaluation (Figure 4A, lower panel) showed normal retinal appearance in wild-type mice but evident disruption at the RPE layer and CNV induction in the retinas of Hcy-injected wild-type mice. Blocking GLUT1 by WZB117 markedly improved retinal structure and CNV induction after Hcy injection, and vascular leakage was further confirmed by determining the albumin leakage in the retinas using western blot analysis following perfusion with PBS solution and as previously described in our publications [9,11,16,17,37]. Western blot data analysis and quantification showed a significant increase in albumin leakage in Hcy-injected mice retina compared to the non-injected mice retina, and the leakage was decreased by WZB117 (Figure 4B), suggesting that blocking GLUT1 could rescue RPE cells, restore the BRB, prevent retinal leakage induced by HHcy, and reduce CNV induction.

### 2.4. Evaluation of Homocysteine-Induced Glycolysis by Seahorse Analysis 

**Figure 5:**
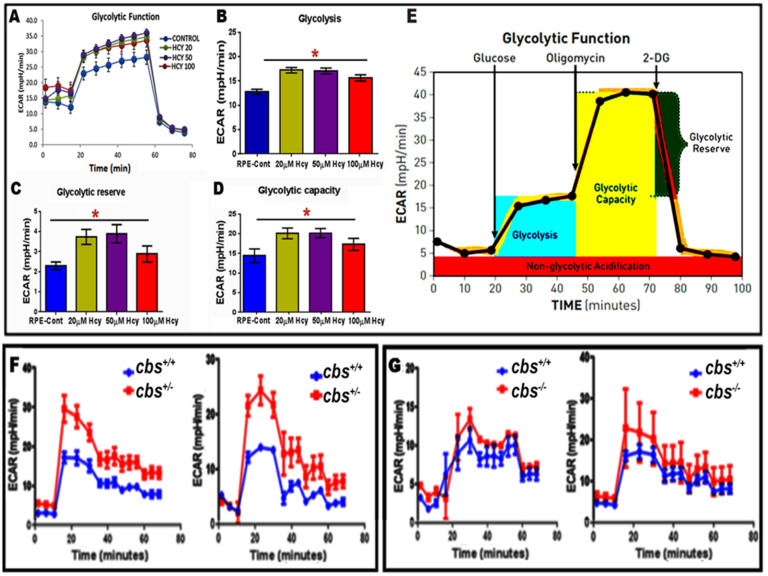
Seahorse analysis evaluation of glycolysis under HHcy. (**A**) ECAR was measured with an XFe96 extracellular flux analyzer in ARPE cells treated with different concentrations of Hcy (20, 50, and 100 μM), showing that ECAR was significantly elevated in Hcy-treated ARPE cells compared to control untreated cells. (**B**) ECAR representative analysis showing significant elevation of glycolysis in Hcy-treated ARPE cells compared to control untreated cells. (**C**) ECAR glycolytic reserve analysis showing significant elevation of glycolysis reserve in Hcy-treated ARPE cells compared to control untreated cells. (**D**) ECAR glycolytic capacity analysis showed significant glycolysis capacity elevation in Hcy-treated ARPE cells compared to control untreated cells. (**E**) Diagram showing the stages of glycolysis as evaluated in RPE cells (**A**–**D**) and mice retina (**F**,**G**). (**F**,**G**) Replicate of ECAR measured with an XFe96 extracellular flux analyzer for retinas isolated from mice model of elevated Hcy (wild-type *cbs^+/^*^+^, *cbs*^+/−^ and *cbs*^−/−^), showing activation of glycolysis parameters in both the *cbs^+/−^* and *cbs^−/−^* compared to wild-type *cbs^+/^*^+^. * *p* < 0.05 as compared to RPE-control.

Seahorse analysis was performed to evaluate glycolysis both in vitro and in vivo using ARPE-19 cells treated with different concentrations of Hcy (20, 50, and 100 μM, representing mild, moderate, and severe HHcy) and fresh retinas from the mice with Hcy (wild-type *cbs^+/+^* mice and *cbs^+/−^* and *cbs^−/−^* mice, representing mild/moderate and severe HHcy). Hcy treatment significantly increased glycolytic reserve (which indicates the capability of a cell to respond to an energetic demand and how close the glycolytic function is to the cell’s theoretical extreme) and glycolytic capacity (which is a measure of the maximum rate of conversion of glucose to pyruvate or lactate that can be achieved intensely by a cell) as shown in Figure 5A–D. Furthermore, seahorse analysis revealed activation of glycolysis in the retinas of mice with HHcy with significant activation of both glycolytic reserve and capacity in *cbs^+/^* and *cbs^−/−^* mice Figure 5F,G. The presented data shows strong evidence that HHcy induces a metabolic shift from mitochondrial respiration to aerobic glycolysis in retinal RPE cells, utilizing a modified cellular metabolism known as the Warburg effect, which is widely acknowledged in cancer cells, and in which energy metabolism relies mainly on glycolysis.

### 2.5. Inhibition of Nmdar Protects RPE Cells and Inhibits Hcy-Induced Activation of Glycolysis

**Figure 6:**
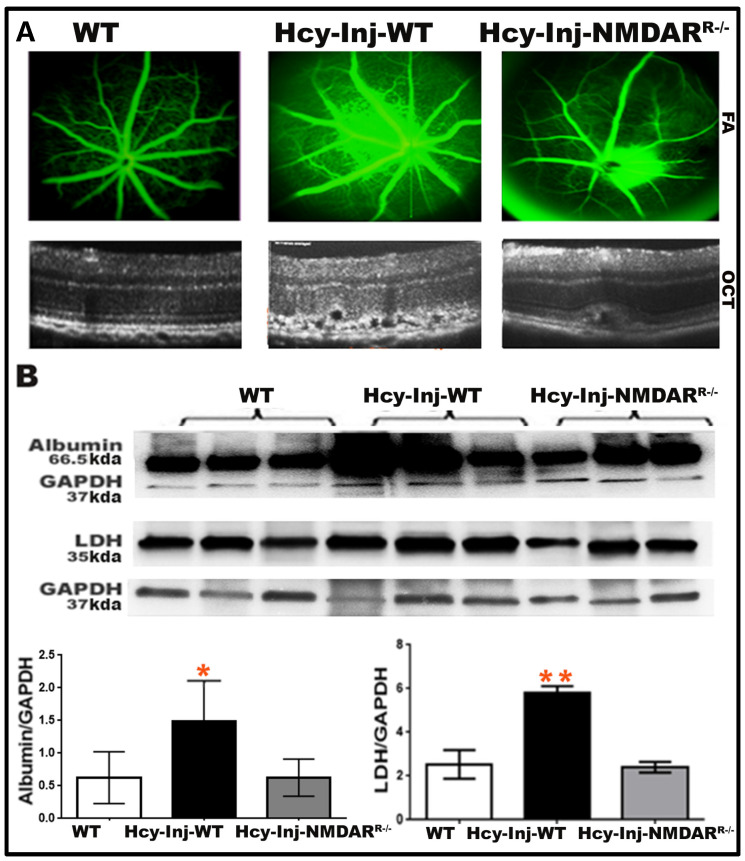
Deletion of NMDAR in RPE cells protected the retina from the harmful effect of Hcy on BRB, CNV induction, and glycolysis activation. (**A**) FA and OCT evaluation of living mice retinas after 72 h after the intravitreal injection of Hcy in WT and *NMDAR^R −/−^* compared to WT-control non-injected mice. Hcy injection induced BRB dysfunction with increased fluorescein leakage (upper panel) and induced choroidal neovascularization while knocking down NMDAR in (*NMDAR^R −/−^*), protecting the retina from the Hcy’s harmful effect and reducing the CNV induction (lower panel). (**B**) Western blot analysis to evaluate albumin leakage and glycolytic enzyme LDH in mice retinas after perfusion of mice with PBS solution. Reduced vascular leakage and glycolysis after blocking NMDAR were confirmed by measuring the albumin leakage and LDH enzyme. Hcy-injection significantly increased albumin leakage and LDH levels in mice eyes, but markedly reduced to normal levels by knocking down NMDAR (in *NMDAR^R−/−^*). GAPDH was used as a loading control. Scale bar: 20 μm. * *p* < 0.05 and ** *p* < 0.01 as compared to wild-type and Hcy-injected *NMDAR^R^^−/−^* mice.

Recently, we reported that NMDAR activation is an underlying mechanism for Hcy in the AMD retina and that blocking NMDAR both by pharmacologic inhibition (MK801) or genetic inhibition of NMDAR in RPE cells (*NMDAR^R−/−^* mouse) restored RPE barrier function and protected the retina from the HHcy-induced choroidal neovascularization [11]. To further understand whether Hcy activation of GLUT1 and metabolic shift to glycolysis is due to the direct effect of Hcy or via activation of the NMDAR, we performed another set of in vivo experiments using three groups of mice at age ~6–8 weeks old (wild-type mice without injection compared to wild-type and *NMDAR^R−/−^* mice after 72 h of intravitreal injection of Hcy (200 μM)). Then, mice were subjected to FA and OCT evaluation (Figure 6). The imaging evaluation in living mice confirmed what we previously reported [11]. The FA examination showed increased fluorescein leakage in Hcy-injected wild-type mice compared to wild-type control mice suggesting decreased retinal vessel integrity and disturbed BRB by Hcy. At the same time, the genetic inhibition of NMDAR by knocking down NMDAR in RPE cells (*NMDAR^R−/−^*) restored BRB and decreased fluorescein leakage (Figure 6A, upper panel). Furthermore, OCT evaluation showed normal retinal appearance in wild-type mice. Still, noticeable disruption at the RPE layer and CNV induction in the retinas of Hcy-injected wild-type mice, knocking down NMDAR in RPE cells (*NMDAR^R−/−^*), was able to improve the retinal structure and the CNV induction after Hcy injection (Figure 6A, lower panel).

Western blot analysis was used to evaluate retinal vascular leakage by assessing albumin and assessing glycolytic enzymes LDH to validate the involvement of NMDAR in the Hcy-induced metabolic shift to glycolysis after retinal perfusion with PBS solution and as we previously described [9,11,16,17,37]. The analysis showed a significant increase in albumin leakage in Hcy-injected mice retina compared to the non-injected mice retina. The leakage was significantly decreased in Hcy-injected *NMDAR^R−/−^*. Additionally, Hcy-significantly increased LDH expression and blocking of NMDAR was able to restore the LDH back to normal levels in *NMDAR^R−/−^* mice retina (Figure 6B).

**Figure 7:**
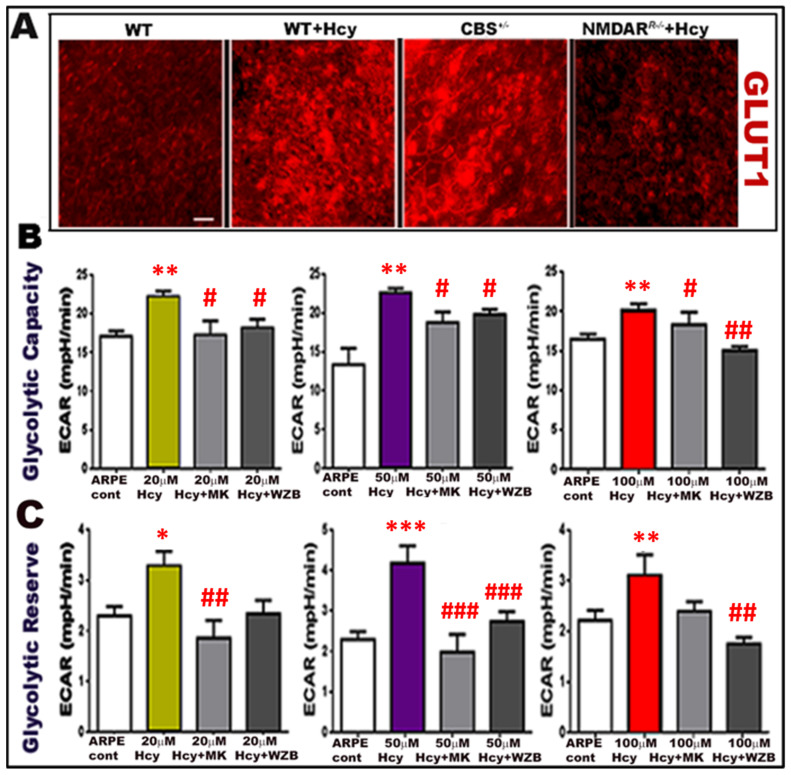
Deletion of NMDAR in RPE cells decreased Hcy-induced activation of glycolysis. (**A**) immunofluorescence staining for RPE flat mounts isolated from mouse retinas after one week of intravitreal injection of Hcy in WT and *NMDAR^R−/−^* compared to WT-control non-injected mice and mice with HHcy (*cbs^+^*^/*−*^) stained with the marker for GLUT1 (red). Hcy injection induced activation of GLUT1, which was more evident in both mice models of HHcy (*cbs^+/−^* mice and Hcy-injected WT mice), while knocking down NMDAR (in *NMDAR^R−/−^*) was able to reduce the activation of GLUT1 induced by Hcy-injection. (**B**) Glycolytic capacity and (**C**) Glycolytic reserve evaluated by seahorse analysis for ARPE-19 cells treated with different concentrations of Hcy (20, 50, and 100 μM) in the presence /or absence of NMDAR inhibitor MK801or GLUT1 inhibitor WZB117. * *p* < 0.05, ** *p* < 0.01, and *** *p* < 0.001 (Hcy treated group compared to ARPE-control group) while ^#^ *p* < 0.05, ^##^ *p* < 0.01, and ^###^ *p* < 0.001 (Hcy + MK treated RPE and Hcy + WZB treated RPE compared to Hcy treated group) for all concentrations.

The second set of experiments was conducted after using the same group’s mice and adding the *cbs^+/−^* mice group. RPE flat-mounts were isolated from mouse retinas one week after intravitreal injection of Hcy (wild-type and *NMDAR^R−/−^* mice). RPE flat-mounts were prepared per our published method [9,15,38] and stained with immunofluorescence stain for GLUT1 (red). Mounts show that Hcy injection-induced activation of GLUT1 in RPE cells was more evident in the two mice models of HHcy (*cbs^+/−^* mice and mice injected intravitreally with Hcy) compared to wild-type control mice. Deletion of NMDAR in RPE cells (*NMDAR^R −/−^* mice) mitigated Hcy-induced activation of GLUT1 (Figure 7A). Finally, an in vitro experiment was performed to evaluate glycolysis by seahorse analysis in ARPE-19 cells treated with different concentrations of Hcy (20, 50, and 100 μM) in the presence/absence of NMDAR blocker, MK801(25 μM), or GLUT1 inhibitor WZB117 (10 μM)). Hcy-treatment of RPE cells significantly increased glycolytic capacity and glycolytic reserve in all concentrations, whereas blocking NMDAR or GLUT1 was able to reduce Hcy-induced activation of glycolysis back to normal average level as observed in control untreated ARPE-19 cells (Figure 7B,C). The presented data suggest that HHcy induces AMD features and metabolic shifts in RPE cells and confirms the involvement of NMDAR in this mechanism. 

## 3. Discussion

Accumulating evidence is supporting the notion that glycolysis may contribute to age-related alterations of the choroidal microvasculature [27]. Kanako et al. found that the lactate–pyruvate ratio was significantly increased in AMD patients, suggesting increased glycolysis and impairment of mitochondrial metabolism in AMD patients [27]. However, the impact of the glycolytic pathway on the progression of AMD has not been deeply studied yet. The current study presents shreds of evidence suggesting that elevated Hcy activates aerobic glycolysis using the Warburg effect as a possible mechanism of BRB dysfunction, Hcy-induced features of AMD, and CNV induction. Previously, we reported that Hcy produced retinal changes with AMD-like features in the RPE with BRB dysfunction and induced CNV [9]. 

RPE cells are dependent mainly on oxidative phosphorylation, while the photoreceptor cells are more dependent on glycolysis [39]; thus, most of the glucose in the outer retina is metabolized via aerobic glycolysis [40]. Over aerobic glycolysis photoreceptor cells, glucose is converted to lactate, which is transported to RPE cells where it can be converted to pyruvate (by the action of LDH), which fuels mitochondria for oxidative phosphorylation [41]. Moreover, the lactate made in the photoreceptor cells is correspondingly transported to Müller cells where it is converted to pyruvate and fuels the mitochondria for oxidative phosphorylation as well. Therefore, there is a metabolic ecosystem that exists between RPE, photoreceptor, and Müller cells [39].

The glycolysis process involves glucose metabolism and the generation of adenosine triphosphate (ATP). The glycolytic pathway yields pyruvate and lactate from glucose [42]. In the presence of oxygen, acetyl-CoA is produced from pyruvate and then oxidized via the Krebs cycle in the mitochondria. However, the lactate dehydrogenase (LDH) enzyme converts pyruvate to lactate [43,44]. This means that lactate production is increased under hypoxic conditions in the tissues. Higher lactate level leads to several functional disorders [45,46,47]. 

The increased lactate production in RPE is associated with visual dysfunction and may contribute to the pathogenesis of AMD [48]. 

Elevated glucose uptake, glycolysis rate, and decreased respiration, leading to lactate production, is known as the Warburg effect. The Warburg effect is observed in fetal cells, rapidly proliferating tumor cells, and retinal cells [49,50].

Hypoxia-inducible factor (HIF) plays a controversial role in glycolysis. HIF was reported in many studies to be a key contributor to glycolysis. Its activation stimulates the expression of glycolytic transporters and enzymes supporting a high rate of aerobic glycolysis [51,52,53,54]. It has been also reported to play an essential role in the Warburg effect [55]. Therefore, its activation is behind the hypoxic metabolic switch in RPE cells. However, other studies addressed the reverse possibility of metabolic control of HIF-1 and suggested that the increased glycolysis rate results in increased lactate production, inhibiting prolylhydroxylase 2 activity and inducing the activity of HIF-1α, with subsequent up-regulation in the activity of many genes involved in angiogenesis, including vascular endothelial growth factor (VEGF) [56].

Previously, we reported Hcy-induced retinal hypoxia in a mouse model of HHcy (*cbs^−/−^* and *cbs^+/−^* mice) [10,13], activation of HIF-1α and VEGF in RPE cells under HHcy with subsequent stimulation of angiogenesis, and finally RPE barrier dysfunction and induction of choroidal neovascularization [11]. 

In this study, we assessed the effect of HHcy on the glycolysis pathway and lactate production in RPE cells (by evaluating the level of Lactate dehydrogenase enzyme) as a mechanism for the pathogenesis of AMD. 

Our results showed that HHcy significantly increased the expression of GLUT1, the primary transporter for glucose receptors in RPE cells, which resulted in an increase in glucose uptake and production of lactate. We further evaluated levels of enzymes involved in glycolysis in RPE cells such as hexokinase enzyme (a key enzyme for glycolysis), and lactate dehydrogenase (the enzyme that converts pyruvate to lactate). HHcy significantly increased the levels of LDH and HK1 glycolytic enzymes. These results suggest that elevated Hcy increases glucose consumption in the RPE cells via activated glycolysis with subsequent increases in cellular acidity and in RPE cells, leading to activation of angiogenesis and age-related features in the retina. 

To confirm our results, seahorse analysis was used in this study to measure the glycolysis rate in the RPE cells treated with Hcy and in the mice retina using the *cbs^+/−^* and *cbs^−/−^* mice (mice with HHcy). The seahorse analysis results confirmed that HHcy significantly increased the glycolysis rate in RPE cells. Moreover, the GLUT1 inhibitor significantly decreased the glycolysis rate in RPE cells by blocking glucose uptake. Likewise, imaging evaluation in a living mouse by FA and OCT and albumin leakage evaluation showed that blocking GLUT1 improved retinal structure and decreased HHcy-induced CNV and vascular leakage after Hcy-intravitreal injection in wild-type mice.

Therefore, GLUT1 inhibition might be a helpful treatment to halt the HHcy-induced Warburg effect in RPE cells, and thus GLUT1 inhibitors might be a helpful therapeutic avenue to protect against the development of AMD.

Previously, we reported that HHcy increased NMDAR expression in retinal endothelium [17] and RPE cells [11]. We concluded that Hcy activation of the NMDA receptor causes Hcy-induced AMD-like features with alteration of the RPE (structure and function) and CNV induction [11]. 

In this study, we proposed that HHcy-induced NMDAR activation in RPE cells plays a role in the HHcy-induced Warburg effect in RPE cells. To verify that, we assessed the impact of NMDAR inhibition using pharmacological inhibition (MK801) on glycolysis rate using seahorse analysis. The results showed that MK801 significantly decreased the glycolysis rate in RPE cells. Furthermore, our results showed that deletion of NMDAR in RPE cells (genetic inhibition) improved retinal morphology, decreased retinal albumin leakage, decreased CNV induction, and significantly decreased the glycolytic enzyme LDH in mouse retina. The presented results demonstrate strong evidence that NMDR activation in RPE cells plays a role in HHcy-induced activation of glycolysis in RPE cells, and its inhibition might protect the retina from BRB dysfunction and AMD pathology.

**Figure 8:**
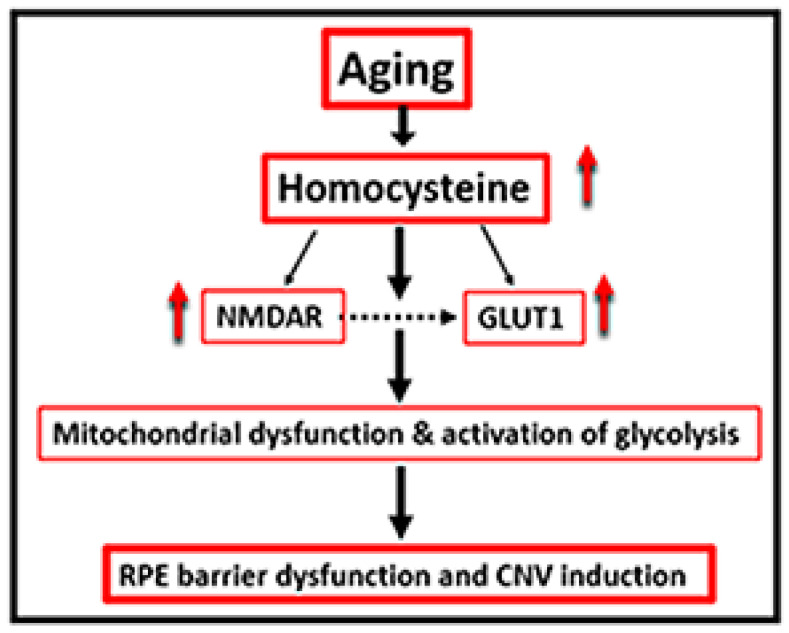
Conclusion for our hypothesis about the role of homocysteine in AMD induction via stimulating NMDAR and GLUT1 producing RPE barrier dysfunction and CNV.

In conclusion, the presented data from the current study show strong evidence that the Warburg effect, with the mitochondrial metabolic shift to glycolysis in RPE cells, might play a vital role in HHcy-induced AMD; thus, targeting the glycolytic pathway or NMDAR provides a valuable target for AMD treatment and insight into a mechanism conducive to novel drug discovery (Figure 8).

## 4. Materials and Methods

### 4.1. Animals

Mouse with HHcy: Breeding pairs of Cystathionine β-synthase (*cbs^+/−^)* mice (B6.129P2-Cbstm1Unc/J; Jackson Laboratories, Bar Harbor, ME, USA) were used to establish colonies of heterozygous (*cbs*^+/*−*^) with one copy of *CBS* or homozygous (*cbs^−^*^/*−*^) and with no copies of *cbs* enzyme and wild-type (*cbs*^+/+^). Consequently, the *cbs*^+/*−*^ mice have mild to moderate elevation of plasma Hcy level about 4 to 7-fold increase, while the *cbs^−^*^/*−*^ mice represent severe HHcy with about a 30-fold increase in plasma Hcy. *cbs^−^*^/*−*^ mice have severe growth retardation and short life span of ~5 weeks. Histological examination showed that the hepatocytes of homozygotes were enlarged, multinucleated, and filled with microvesicular lipid droplets. Cbs mice have been used as a model of HHcy in our publication and those of others [9,10,11,13,14,15,16,17,37,57].

Mouse with deletion or inhibition of NMDAR in RPE: A mouse deficient in NMDAR in RPE cells (*NMDAR^R−/−^)* was generated in our lab by backcrossing C57BL/6-Tg (BEST1-cre) 1Jdun/J mice (Jackson Lab) that express Cre recombinase under the control of the human bestrophin 1 (*BEST*) promoter with floxed NR1 mice to study the role of RPE NMDAR in Hcy-induced AMD. Our previous publication mentioned the dilated description of mouse generation and genotyping in detail [11]. Mice homozygous for this allele are viable, fertile, and do not display any gross physical or behavioral abnormalities. 

Hcy intravitreal injection in the eyes of mice was described in our previous publications [9,11,16,17,37]. We injected, intravitreal, 1 μL volume in the eye. We dissolved 10X stock solution of L-Homocysteine thiolactone hydrochloride (Sigma-Aldrich, Louis, MO, USA) in distilled water with ultimate preparation of a working solution of 200 μM vitreal concentration of Hcy-thiolactone, and 1 μL of this working solution was injected. 

All experiments were conducted according to Oakland University guidelines and followed the ARVO Statement for Use of Animals in Ophthalmic and Vision Research and the Public Health Service Guide for the Care and Use of Laboratory Animals (Department of Health, Education, and Welfare publication, NIH 80-23). All animals were kept in plastic cages, allowed to eat and drink ad libitum, and were exposed to 12-h light/12-h dark-light cycles and 22 to 24 °C temperature. IACUC PROTOCOL NUMBER:2022-1160 (21063).

### 4.2. Cell Culture

The human retinal pigmented epithelial cell line (ARPE-19) was purchased from American Type Culture Collection (ATCC, Manassas, VA, USA). The passages 6–15 were used in our experiments and cultured in DMEM/F-12 growth medium (Thermo Scientific, Wyman, MA, USA) supplemented with 10% fetal bovine serum (FBS) and 1% penicillin/streptomycin. When the cells reach 80–90% confluency, the cells were cultured in serum-free media overnight and then treated with Hcy (20 or 50 or 100 μM) or vehicle for 24 h. The cells then were harvested for additional assays.

### 4.3. Isolation and Culture of Primary Retinal Pigment Epithelium (RPE)

RPE cells were isolated from wild-type, *cbs^+/−^* and *cbs^−/−^* mice (~3 weeks old) as previously published [11,15,58]. In brief, enucleated mouse eyes were washed with betadine solution, then sterile Hank’s balanced salt solution (HBSS). After that, the muscles and connective tissues surrounding the eyeballs were removed and the eyes were transferred to a cold (DMEM: F12) medium, supplemented with 25% FBS, 0.1 mg/mL gentamicin, 100 U/mL penicillin, and 100 μg/mL streptomycin. Then eyes were moved to HBSS containing collagenase (19.5 U/mL) and testicular hyaluronidase (38 U/mL) and incubated at 37 °C for 40 min. After that eyes were transferred to HBSS containing 0.1% trypsin (pH 8) and incubated for 50 min at 37 °C. Then, eyes were set in a dish containing DMEM: F12 medium for at least 30 min at 4 °C. This was followed by dissection of the eyes, isolation of the RPE cells, then centrifugation for 10 min at 1200× *g*. The RPE cells were suspended and cultured in DMEM/F12 at 37 °C.

### 4.4. Western Blot Analysis

GLUT1 and glycolytic enzymes; hexokinase 1 (HK-1) and lactate dehydrogenase (LDH) in RPE cells and albumin in mice retina were detected by western blot analysis. After treatment of ARPE-19 with Hcy, the media were removed and RIPA buffer supplemented with 1:100 (*v*/*v*) of proteinase/phosphatase inhibitor cocktail was added and cells were lysed. Then, the mouse retina and primary RPE cells were lysed in the RIPA buffer supplemented with a proteinase/phosphatase inhibitor. The tissue homogenate and cell lysates were centrifuged at 4 °C and 12,000× *g* for 30 min. The protein concentration was measured by BCA protein assay (Thermo Scientific, Waltham, MA, USA), then an equal amount of protein was boiled in Laemmli sample buffer. Samples were subjected to gel electrophoresis on sodium dodecyl sulfate-polyacrylamide gel (SDS-PAGE) and the protein was blotted to nitrocellulose membranes. After that, membranes were blocked by 5% milk solution and then incubated with VEGF (Thermofisher, MA, USA, Cat. #5-13182), HIF-1α (Abcam, Waltham, MA, USA, Cat. # ab82832), NMDAR1 (Cell Signaling, Danvers, MA, USA, Cat. # 5704S), NMDAR2A (Cell Signaling, Danvers, MA, USA, Cat. # 4205s), NMDAR2B (Cell Signaling, Danvers, MA, USA, Cat. # 4207s), Albumin (Bethyl, TX, USA), βactin (Cell Signaling, Danvers, MA, USA, Cat. #937215), and GAPDH (Sigma-Aldrich, St. Louis, MO, USA). Suitable peroxidase-conjugated secondary antibodies were added to the blots and incubated then the blot was visualized with the enhanced chemiluminescence (ECL) western blot detection system (Thermo Scientific). The optical density of the bands was determined by ImageJ software, National Institutes of Health, USA. 

### 4.5. Immunofluorescent Assessment

Primary mouse cells, ARPE19 cells, and RPE retinal flat-mounts were subjected to immunofluorescent staining to detect GLUT-1 and Glycolytic enzymes (HK-1 and LDH). First, ARPE19 and primary RPE cells were fixed in 4% paraformaldehyde for 10 min then washed with PBS and blocked with Power Block (BioGenex, Fremont, CA, USA, Cat. # BS-1310-25) for one hour. Then cells were incubated with anti-HK-1 (Abcam, MA, USA, Cat. # ab82832), anti-GLUT-1 (Cell Signaling, Danvers, MA, USA. Cat. # 5704S), and anti-LDH (Thermofisher, MA, USA, Cat. #5-13182) for three h at 37 °C. Then, cells were washed with PBS containing 0.3% Triton-X 3 times. After washing, cells were incubated with appropriate secondary antibodies (Alexa fluor and Texas red avidin, Invitrogen, Eugene, Oregon) and coverslipped with DAPI (Sigma-Aldrich Chemical Corp., St. Louis, MO, USA). Images were captured by a fluorescent microscope (Carl Zeiss, Göttingen, Germany) with a high-resolution camera using Zeiss Axiovision digital image processing software (version 4.8). GLUT-1 expression was evaluated in RPE flat-mounts isolated from mice injected intravitreally with Hcy (WT and *NMDARR^−/−^* mice). RPE flat-mounts were conducted per our published methods [11,15].

### 4.6. Seahorse Analysis for Glycolytic Function Assessment

Glycolysis stress test kit and Seahorse XFe96 (Agilent, Santa Clara, CA, Cat. # 103020-100) were used to evaluate glycolysis. Briefly, ARPE19 cells were seeded in the Seahorse cell culture plate in a DMEM/F-12 medium. The cartridges were hydrated in a non-CO2 incubator at 37 °C overnight. ARPE19 cells were treated with Hcy at (20 or 50 or 100 μM) in the presence/absence of NMDR inhibitor (25 μM MK801) or GLUT-1 inhibitor (μM WZ117) or vehicle for 24 h. For the Glycolysis stress test assay, cells were glucose-starved in an XF assay medium containing 2 mM glutamine iCO2-free incubator at 37 °C for one hour. Extracellular acidification rates (ECAR) were measured by first injecting glucose (FWC 10 mM) and the cells catabolize glucose into pyruvate via the glycolysis pathway, producing ATP, NADH, water, and protons. The extrusion of protons into the surrounding medium led to a sudden increase in ECAR, defined as basal glycolytic capacity. Then, oligomycin (FWC 1 μM) was injected to shift energy production to glycolysis by inhibiting mitochondrial ATP synthesis, and the sharp increase of ECAR after oligomycin injection indicated the level of glycolytic capacity. Finally, 2-deoxyglucosee (2-DG) (FWC 50 mM), a glucose analog, which inhibited glycolysis through competitive binding to glucose hexokinase, was injected into the wells. The decrease in ECAR confirmed that the ECAR produced in the experiment was caused by glycolysis. The gap between glycolytic capacity and glycolysis was defined as a glycolytic reserve. ECAR, prior to glucose injection, was referred to as non-glycolytic acidification, and this may be due to other processes in cells. Glycolysis, glycolytic capacity, glycolytic reserve, and non-glycolytic acidification were calculated according to the manufacturer’s instructions. Data were represented in mpH/min.

### 4.7. Data Analysis

Results were expressed as mean ± SD. The differences between experimental groups were assessed using the two-tailed test or one-way analysis of variance (ANOVA). If the statistical differences were detected using ANOVA, Tukey’s post hoc test was conducted to determine which groups differed. Statistical significance was considered at a confidence level of *p* < 0.05. 

### 4.8. Optical Coherence Tomography (OCT) and Fluorescein Angiography (FA)

To evaluate the fluorescein leakage and retinal morphology after intravitreal injection of Hcy into wild-type and *NMDAR^−/−R^* mice, OCT and FA were performed according to our published methods with some modifications [9,11,16,17,37]. Briefly, the mice were injected intravitreally with Hcy (200 μM) and, after 72 h, OCT and FA were conducted. The mice were subjected to anesthesia using 2% isoflurane and the eye pupils were dilated by 1% tropicamide eye drop. Then, each mouse was placed and imaged on the imaging platform of the Phoenix Micron III retinal imaging microscope supplemented with an OCT imaging device (Phoenix Research Laboratories, Pleasanton, CA, USA). Lubricant gel was added to the eye to keep it moist during imaging. For FA, 10% fluorescein sodium (Apollo Ophthalmic, Newport Beach, CA, USA) was injected into the mice (10 to 20 μL, IP) followed by rapid acquisition of fluorescent images succeeded for ~5 min. Fluorescein leakage establishes as indistinct vascular borders gradually progressing to diffusely hazy green fluorescence.

## Data Availability

The data presented in this study are available on request from the corresponding author.
